# Endoscopic and clinical features of gastric emphysema

**DOI:** 10.1038/s41598-024-52633-z

**Published:** 2024-01-25

**Authors:** Masaya Iwamuro, Ryuta Takenaka, Tatsuya Toyokawa, Masahide Kita, Takao Tsuzuki, Masao Yoshioka, Tatsuhiro Gotoda, Shotaro Okanoue, Minoru Matsubara, Chihiro Sakaguchi, Motoyuki Otsuka

**Affiliations:** 1 Department of Gastroenterology and Hepatology, Okayama University Graduate School of Medicine, Dentistry, and Pharmaceutical Sciences, 2-5-1 Shikata-cho, Kita-ku, Okayama, 700-8558 Japan; 2 Department of Internal Medicine, Tsuyama Chuo Hospital, 1756 Kawasaki, Tsuyama, Okayama 708‑0841 Japan; 3 Department of Gastroenterology, National Hospital Organization Fukuyama Medical Center, 4-14-17 Okinogami-cho, Fukuyama, Hiroshima 720-8520 Japan; 4 Department of Internal Medicine, Okayama City Hospital, 3-20-1 Kitanagase Omote-cho, Kita-ku, Okayama, Okayama 700‑8557 Japan; 5 Department of Internal Medicine, Japanese Red Cross Society Himeji Hospital, 1-12-1 Shimoteno, Himeji, Hyogo 670-8540 Japan; 6 Department of Internal Medicine, Okayama Saiseikai General Hospital, 2-25 Kokutai-cho, Kita-ku, Okayama, 700-8511 Japan; 7 Department of Gastroenterology and Hepatology, Kurashiki Central Hospital, 1-1-1 Miwa, Kurashiki, Okayama 710-8602 Japan; 8 Department of Gastroenterology, Mitoyo General Hospital, 708 Himehama, Toyohama-cho, Kan-onji, Kagawa, 769-1695 Japan; 9 Department of Gastroenterology, Sumitomo Besshi Hospital, 3-1, Ojicho, Niihama, Ehime 792‑8543 Japan; 10 Department of Endoscopy, National Hospital Organization Shikoku Cancer Center, Kou 160, Minamiumemotomachi, Matsuyama, 791-0280 Japan

**Keywords:** Gastrointestinal diseases, Stomach diseases

## Abstract

Gastric emphysema is characterized by the presence of intramural gas in the stomach without bacterial infection. Due to its rarity, most reports on gastric emphysema have been limited to single-case studies, and this condition’s clinical and endoscopic features have not been thoroughly investigated. In this study, we analyzed 45 patients with gastric emphysema from 10 institutions and examined their characteristics, endoscopic features, and outcomes. The mean age at diagnosis of gastric emphysema in our study population (35 males and 10 females) was 68.6 years (range, 14–95 years). The top five underlying conditions associated with gastric emphysema were the placement of a nasogastric tube (26.7%), diabetes mellitus (20.0%), post-percutaneous endoscopic gastrostomy (17.8%), malignant neoplasms (17.8%), and renal failure (15.6%). Among the 45 patients, 42 were managed conservatively with fasting and administration of proton pump inhibitors. Unfortunately, seven patients died within 30 days of diagnosis, and 35 patients experienced favorable recoveries. The resolution of gastric emphysema was confirmed in 30 patients through computed tomography (CT) scans, with a mean duration of 17.1 ± 34.9 days (mean ± standard deviation [SD], range: 1–180 days) from the time of diagnosis to the disappearance of the gastric intramural gas. There were no instances of recurrence. Endoscopic evaluation was possible in 18 patients and revealed that gastric emphysema presented with features such as redness, erosion, coarse mucosa, and ulcers, with fewer mucosal injuries on the anterior wall (72.2%), a clear demarcation between areas of mucosal injury and intact mucosa (61.1%), and predominantly longitudinal mucosal injuries on the stomach folds (50.0%). This study is the first English-language report to analyze endoscopic findings in patients with gastric emphysema.

## Introduction

Gastric emphysema, also known as gastric pneumatosis, is a medical condition in which gas accumulates within the layers of the stomach wall caused by non-infectious mechanisms^1,2^. The introduction of air or gas into the stomach wall results from medical procedures, such as endoscopic gastrostomies, gastric mucosal damage following contact with a feeding tube, or other underlying medical conditions. This gas accumulation can lead to a characteristic radiological appearance resembling air- or gas-filled pockets in the stomach wall. Emphysematous gastritis is another medical condition characterized by the presence of gas within the stomach wall, caused by the invasion of gas-producing bacteria into the stomach wall^3–5^. These bacteria produce gas as a by-product of their metabolic processes, leading to the accumulation of gas beneath the stomach lining. Emphysematous gastritis is a form of infectious or phlegmonous gastritis and is typically associated with severe inflammation and tissue damage, leading to serious and occasionally life-threatening conditions.

Intramural gas in the stomach cannot be directly identified through esophagogastroduodenoscopy, thereby necessitating the use of radiological modalities, such as computed tomography (CT), to diagnose gastric emphysema and emphysematous gastritis. Consequently, when gastric emphysema or emphysematous gastritis is suspected during endoscopy, expeditious CT is imperative. However, owing to its rarity, the endoscopic features of gastric emphysema and emphysematous gastritis have not been sufficiently investigated. Therefore, in the current study, we investigated 46 patients in whom intramural air in the stomach was detected on CT scans from multiple institutions and analyzed the endoscopic features of 18 patients in which esophagogastroduodenoscopy was performed. We also investigated the patients’ backgrounds and outcomes.

## Methods

**Figure 1:**
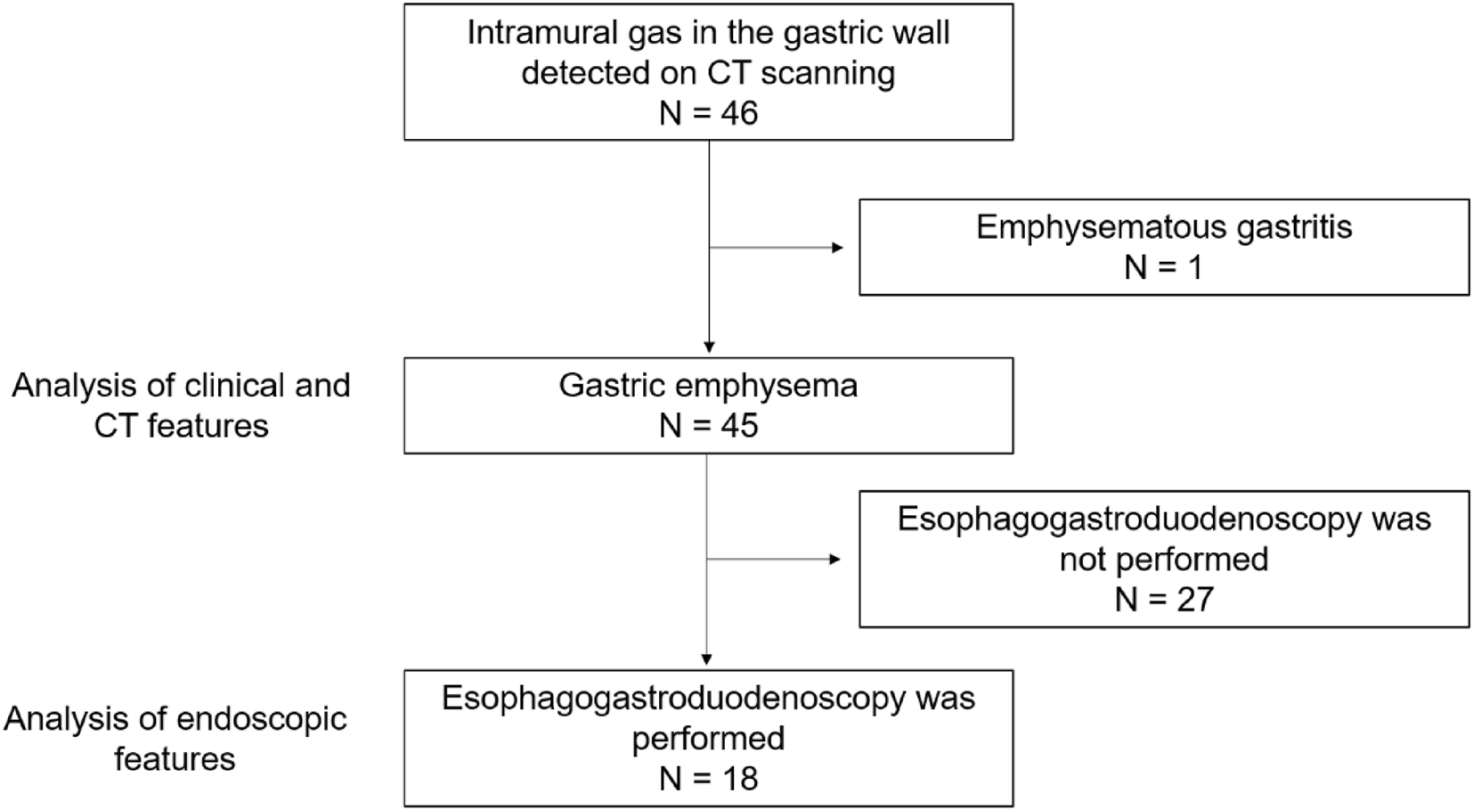
Study flow chart. *CT* computed tomography.

**Figure 2:**
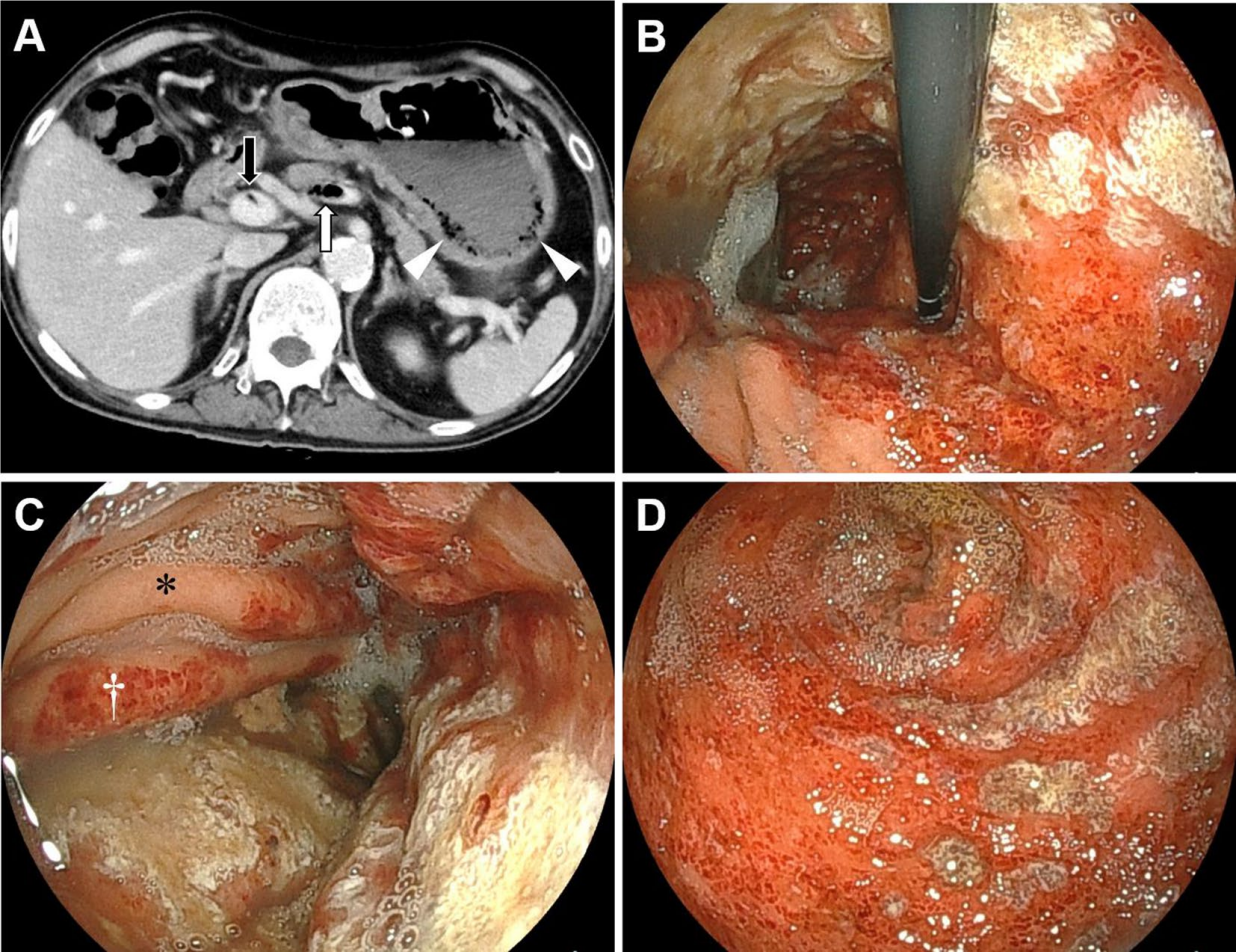
CT and endoscopic images of a 66-year-old male, currently undergoing chemotherapy for esophageal cancer, with a gastrostomy tube placement. The patient was asymptomatic, and during an endoscopic evaluation before esophageal cancer surgery, gastric mucosal injury was noted. On the same day, CT scans (**A**) revealed the presence of gas (white arrow) and thrombosis (black arrow) in the splenic to portal veins. Additionally, a gas-filled appearance was observed within the gastric wall (arrowheads). Esophagogastroduodenoscopy (**B**–**D**; **B**, the gastric fornix and upper body; **C**, gastric body; **D**, gastric antrum) showed diffuse reddish erosions with white exudates extending from the fornix to the antrum. The boundary between the injured and non-injured mucosa was partially distinct (**B**). The anterior wall of the gastric body was intact (**C**, asterisk). Mucosal injury was predominant on the folds (**C**, dagger). *CT* computed tomography.

**Figure 3:**
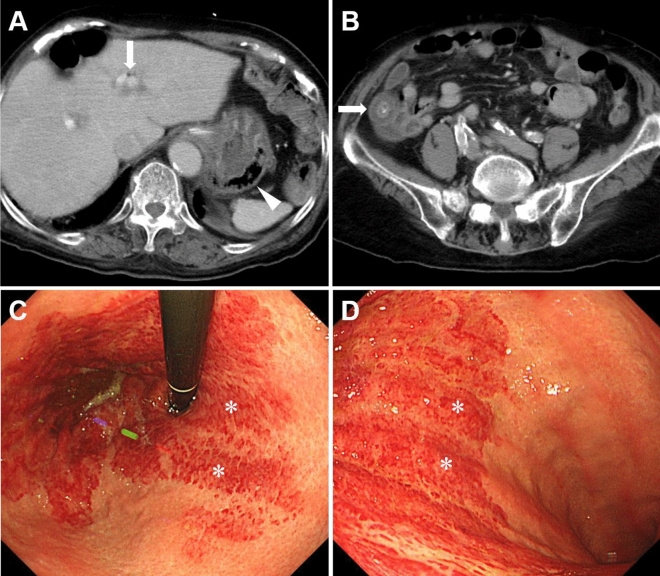
CT and endoscopic images of an 89-year-old female. The patient had a nasogastric tube inserted and a history of cerebral infarctions with internal carotid artery occlusion, chronic atrial fibrillation, aortic valve stenosis, and hypertrophic cardiomyopathy. She presented with a fever, and CT scans revealed portal venous gas (**A**, arrow), intramural gas in the stomach (**A**, arrowhead), and wall thickening of the ascending colon (**B**, arrow). Esophagogastroduodenoscopy (**C**,**D**) showed a circumferential erythematous erosion in the gastric fornix to the upper body. The boundary between the erythematous area and the intact mucosa was clear. The erythematous erosions were predominant on the convex part of the gastric folds and tended to be longitudinal (**C**,**D**, asterisks). *CT* computed tomography.

Patients with radiologically diagnosed gastric emphysema or emphysematous gastritis between November 2008 and August 2023 at the Okayama University Graduate School of Medicine, Dentistry, and Pharmaceutical Sciences and nine collaborating institutions were enrolled in this study (Fig. 1). Gastric emphysema or emphysematous gastritis was diagnosed based on the presence of intramural air in the stomach on CT images (Fig. 2A, arrowheads and Fig. 3A, arrowhead). A subset of the patients examined (5/46) also participated in our previous studies^6–9^. We conducted a retrospective chart review of the patients’ age, sex, underlying diseases, presence or absence of portal venous gas, presence or absence of colorectal wall thickening, treatment, and outcomes. Endoscopic findings were analyzed in patients who underwent esophagogastroduodenoscopy.

This study was approved by the ethics committees of Okayama University Hospital and of the participating institutions. The requirement for written informed consent was waived by the ethics committees of Okayama University Hospital and the participating institutions because of the retrospective nature of the study and the analysis using anonymous clinical data. All investigations were performed in accordance with the relevant guidelines and regulations and were conducted in accordance with the Declaration of Helsinki. Numerical values are presented as means ± standard deviations (SDs).

## Results

Overall, 46 patients with intramural air in the stomach were enrolled from 10 institutions (Fig. 1). One patient (a 58-year-old male) underwent endoscopic submucosal dissection for early gastric cancer. He experienced abdominal pain on the evening after the procedure, and a CT scan revealed gas within the gastric wall. Esophagogastroduodenoscopy was not performed after the onset of the intramural gastric gas. Subsequently, a total gastrectomy was performed, and pathological analysis of the resected specimen revealed phlegmonous inflammation of the gastric wall, indicating emphysematous gastritis. The other 45 patients were diagnosed with gastric emphysema. Initially, we planned to include emphysematous gastritis in our analysis; however, only one case was reported. Therefore, in the present investigation, we focused on the 45 patients with gastric emphysema and analyzed their backgrounds, CT and endoscopic features, and outcomes.

The mean age at diagnosis of gastric emphysema in the 45 patients (35 males and 10 females) was 68.6 years (range, 14–95 years). Underlying conditions that two or more patients had at the onset of gastric emphysema included placement of nasogastric tubes (n = 12, 26.7%), diabetes mellitus (n = 9, 20.0%), post-percutaneous endoscopic gastrostomy (n = 8, 17.8%), malignant neoplasms (n = 8, 17.8%; esophageal cancer, n = 4; gastric cancer, n = 1; rectal cancer, n = 1; hypopharyngeal cancer, n = 1; concurrent gingival, pharyngeal, and esophageal cancer, n = 1), renal failure (n = 7, 15.6%), stroke (n = 5, 11.1%), post-tracheostomy (n = 5, 11.1%), acute enteritis (n = 5, 11.1%), hypertension (n = 5, 11.1%), liver diseases (n = 4, 8.9%; liver cirrhosis, n = 2; alcoholic steatohepatitis, and n = 1; post-liver transplantation due to primary biliary cholangitis, n = 1), perforation of the gastrointestinal tract (n = 3, 6.7%; duodenum, n = 2; esophagus, n = 1), endotracheal intubation without tracheostomy (n = 3, 6.7%), radiotherapy for cancer (n = 3, 6.7%), excessive alcohol consumption (n = 3, 6.7%), ulcerative colitis (n = 2, 4.4%), heart failure (n = 2, 4.4%), sepsis (n = 2, 4.4%), and abdominal aneurysm (n = 2, 4.4%). Furthermore, nine patients had concurrent respiratory or thoracic diseases (20.0%). These included non-infectious chronic lung diseases (n = 4, 8.9%; chronic obstructive pulmonary disease or bronchial asthma), infectious diseases (n = 4, 8.9%; pneumonia or empyema), and intrathoracic injuries (n = 3, 6.7%; hemothorax, pneumothorax, or mediastinal emphysema), with some overlap.

Abdominal pain was the most frequent symptom (n = 13, 28.9%), followed by nausea or vomiting (n = 11, 24.4%), hemorrhage (hematemesis or melena, n = 10, 22.2%), and fever (n = 8, 17.8%). In contrast, 10 patients (22.2%) in whom gastric emphysema was unexpectedly identified during CT scans conducted for unrelated medical conditions, remained asymptomatic. Portal venous gas was identified on CT scans in 30 patients (66.7%) (Fig. 2A, white arrow and Fig. 3A, arrow). Portal vein thrombosis was observed in three patients (6.7%), all of whom had no underlying liver disease. Partial wall thickening of the colorectum was observed in 9 patients (20.0%) (Fig. 3B, arrow).

Three patients underwent surgery for gastric emphysema, and a 91-year-old male underwent endoscopic submucosal dissection for early gastric cancer and developed gastric emphysema the day after the procedure. Surgical resection of the stomach was performed, and the patient recovered. A 67-year-old male patient presented with gastric emphysema, portal venous gas, portal venous thrombosis, and small bowel necrosis after a percutaneous endoscopic gastrostomy. Consequently, the patient underwent gastric and small bowel resections, ultimately leading to the preservation of his life. A 43-year-old male patient presented with abdominal pain, and a CT scan revealed gastric emphysema and free air around the duodenum. Subsequently, the patient underwent laparoscopic closure with omental patching for a duodenal perforation secondary to duodenal peptic ulcers.

Although esophagogastroduodenoscopy was performed in 20 patients, the mucosa of the stomach could not be evaluated in two patients due to significant bleeding. Thus, the endoscopic features of 18 patients were analyzed. Gastric emphysema was characterized by redness, erosion, coarse mucosas, and ulcers (Figs. 2B–D, 3C,D). Fewer mucosal injuries were observed on the anterior wall (n = 13, 72.2%) than on the posterior wall, greater curvature, and lesser curvature. Eleven patients (61.1%) exhibited a distinct demarcation between the site of mucosal injury and the intact mucosa. Nine patients (50.0%) displayed predominantly longitudinal mucosal injuries on the folds of the stomach.

Except for three patients in whom surgery was performed, 42 patients were conservatively treated with fasting and proton pump inhibitors. Some patients also received antibiotics. During the mean observation period of 430 ± 650 days (range, 0–2664 days), 20 patients died, whereas the remaining 25 patients were alive at the last hospital visit. Computed tomography (CT) was conducted on 30 patients following the diagnosis of gastric emphysema for confirming the resolution of gastric emphysema. The duration from diagnosis to the disappearance of the intramural gas in the stomach was 17.1 ± 34.9 days (mean ± SD, range: 1–180 days). None of the patients experienced a recurrence of gastric emphysema.

To identify the factors contributing to the prognosis of gastric emphysema, we compared the characteristics of patients who died within 30 days of diagnosis (Group A, n = 7) with those who survived for ≥ 31 days (Group B, n = 38) (Table 1). The periods from diagnosis of gastric emphysema to death were as follows: on the day of diagnosis (n = 4), one day later (n = 1), two days later (n = 1), and eleven days later (n = 1). The causes of death were non-occlusive mesenteric ischemia (n = 2), esophageal cancer (n = 1), gastric cancer (n = 1), central nervous system malignant lymphoma (n = 1), gastrointestinal perforation and sepsis (n = 1), and mesenteric and superior mesenteric artery embolisms (n = 1). No differences were observed in age (mean ± SD, 77.6 ± 6.8 vs. 66.9 ± 2.9 years), sex (male/female, 4/3 vs. 31/7), white blood cell count (12,771 ± 6330 vs. 12,791 ± 6565/μL), and presence or absence of portal venous gas (present/absent, 6/1 vs. 24/14). Although the difference was not statistically significant, C-reactive protein values tended to be higher in Group A than in Group B patients (11.9 ± 13.0 vs. 7.1 ± 7.5 mg/dL, P = 0.092). Group A exhibited a greater percentage of individuals without symptoms than Group B (57.1% vs. 15.8%, P = 0.034). None of the patients in Group A exhibited colorectal wall thickening, whereas 23.7% of the patients in Group B did (present/absent, 0/7 vs. 9/29). However, this difference was not statistically significant (P = 0.064). Patients in Group A experienced unfavorable outcomes in a short period; therefore, with the majority exhibiting an inferior general status, endoscopic examinations could only be conducted in one case, making it impossible to perform sufficient statistical analysis regarding endoscopic findings, which comprise the presence or absence of fewer mucosal injuries on the anterior wall, a clear demarcation between the areas of mucosal injury and intact mucosa, and predominantly longitudinal mucosal injuries on the stomach folds.

**Table 1:** Comparison of patient characteristics between individuals deceased within 30 days and those surviving beyond 31 days.

	Group A Patients died within 30 days	Group B Patients who survived for more than 31 days	P value
Number of patients	7	38	
Age (years, mean ± SD)	77.6 ± 6.8	66.9 ± 2.9	0.157
Sex			0.172
Male	4	31	
Female	3	7	
White blood cell count (/μL, mean ± SD)	12,771 ± 6330	12,791 ± 6565	0.994
CRP (mg/dL, mean ± SD)	13.1 ± 13.6	7.1 ± 7.4	0.092
Symptoms			0.034
Present	3	32	
Absent	4	6	
Portal venous gas			0.396
Present	6	24	
Absent	1	14	
Colorectal wall thickening			0.315
Present	0	9	
Absent	7	29	

*SD* standard deviation.

## Discussion

In this retrospective analysis, we collected data from 45 patients with gastric emphysema at 10 institutions. Gastric emphysema is an infrequent clinical entity, with the majority of the existing literature predominantly comprising solitary case reports. Consequently, the present investigation is the most extensive study dedicated to an in-depth analysis of patients with gastric emphysema. To our knowledge, this is the first English-language publication to report the endoscopic features of this disease.

In our previous research, we investigated five patients with gastric emphysema encountered within a single medical institution and subsequently reported the clinical features in a Japanese academic journal^9^. By analyzing endoscopic images from the five patients, we observed the following points: (i) the border between the damaged and intact mucosa was clearly defined, (ii) mucosal injury was less likely to occur on the anterior wall, and (iii) in certain patients, mucosal damage exhibited a conspicuous predilection for the convex segments of the gastric folds, as opposed to their concave counterparts. In the present study, analysis of endoscopic images from 18 patients spanning multiple facilities revealed the following prevalence: a tendency towards fewer mucosal injuries on the anterior wall in 72.2% of patients, conspicuous demarcation of mucosal injuries in 61.1%, and occurrence of longitudinal mucosal injuries on the convex portion of the gastric folds in 50.0%. Although these endoscopic characteristics may also be observed in nonspecific gastritis, we believe that understanding these unique features enables diagnosis of gastric emphysema based on endoscopic findings. In one of the 45 patients, esophagogastroduodenoscopy was performed following gastrointestinal bleeding, and the presence of the aforementioned features raised the suspicion of gastric emphysema, leading to a confirmed diagnosis through subsequent CT scans (data not shown). We speculate that the infiltration of gas into the gastric wall, leading to the disruption of its layered structure, induces ischemia and results in mucosal injuries such as erosions, ulcers, and erythema. We also hypothesized that the distinct demarcation between the mucosal injury sites and intact mucosa aligns with the boundary of the detached gastric wall. The reasons for fewer mucosal injuries on the anterior wall and concave regions of the gastric folds are not yet clear. However, it is plausible that anatomical factors, such as a reduced likelihood of exposure to gastric acid in the concave areas and the anterior wall, may be contributing factors.

Gastric emphysema can be classified into three types based on its etiology: traumatic, obstructive, and pulmonary^10–13^. Traumatic-type gastric emphysema involves the migration of gas from the injured mucosa in the stomach caused by peptic ulcers or mechanical damage, such as nasogastric tube contact and gastrostomies. Obstructive gastric emphysema results from gastrointestinal obstruction, which leads to increased gastric pressure and subsequent gas penetration into the gastric wall. Pulmonary-type gastric emphysema occurs when gas originating from the lungs enters the gastric wall due to conditions such as pulmonary emphysema or the rupture of pulmonary bullae. In this study, some patients underwent nasogastric tube placement (26.7%) or gastrostomies (17.8%). Trauma to the gastric mucosa may have induced intramural gas in these patients. Gas may have emanated from the perforated mucosa in the duodenum or esophagus in patients who had gastrointestinal tract perforation (6.7%). The gas also plausibly migrated from the trachea, bronchi, or bronchioles in patients who underwent tracheostomies (11.1%) or endotracheal intubations (6.7%). Notably, 20.0% of patients had concurrent respiratory or thoracic diseases, including infectious and non-infectious chronic lung diseases and intrathoracic injuries. These comorbidities may contribute to the development of pulmonary-gastric emphysema. Irrespective of the possible etiological factors, no recurrence was observed in the patients who survived, prompting our assertion that the treatment strategy should not be amended in accordance with the underlying conditions.

Although patients with severe underlying conditions such as malignant tumors, sepsis, and intestinal ischemia succumbed, most patients survived for ≥ 31 days among those who were conservatively managed (35/42, 83.3%), and the amelioration of gastric emphysema was substantiated 17.1 ± 34.9 days after the diagnosis. These data align with the established consensus that gastric emphysema follows a favorable clinical course and undergoes spontaneous resolution^1,14,15^. We considered the following therapeutic principles for managing gastric emphysema: bowel rest through fasting, intravenous administration of proton pump inhibitors, and use of antibiotics when indicated. Nevertheless, in patients with signs of digestive tract necrosis, such as acidosis, worsening gastric emphysema, ascites, or the emergence of intra-abdominal free air, emergency surgical intervention may be required. Hence, vigilant monitoring of patient conditions is essential throughout the course of conservative treatment.

A comparison of patient characteristics between patients who died within 30 days (Group A) and those who survived for ≥ 31 days (Group B) revealed that the former group exhibited a higher percentage of individuals without symptoms. The reason why a majority of the patients with poorer prognoses (Group A) presented without symptoms remains unknown. One possible reason is that Group A included a significant number of patients whose overall health status might have been compromised such that they were initially unable to express their symptoms. Another possible explanation is that patients in Group A might have had an infection in the gastric lesions, such as emphysematous gastritis, rather than gastric emphysema. Although not statistically significant, a tendency for higher C-reactive protein levels was noted in Group A than in Group B, suggesting these possibilities.

Portal venous gas has historically been considered a relatively infrequent clinical entity predominantly associated with severe intra-abdominal pathologies, notably intestinal necrosis. Nevertheless, in recent years, portal venous gas has been incidentally detected on CT scans and is known to resolve with conservative treatment in a considerable proportion of patients. There have been reports of patients in whom portal venous gas coexists with gastric emphysema in the existing literature^11,16^. The mechanism by which gastric emphysema leads to portal venous gas is postulated to involve direct entry of gas accumulated within the gastric wall into the venous system, eventually reaching the portal vein. Colorectal wall thickening was observed in 20.0% of patients in this study. As colonoscopy was not performed in all patients except for one patient with cytomegalovirus colitis^7^, leaving many aspects regarding the causes of colorectal wall thickening in gastric emphysema and the relationship between the two conditions unclear. Further accumulation of cases is required for a better understanding of this feature.

Gastric emphysema is primarily driven by non-infectious mechanisms, whereas emphysematous gastritis is associated with infections of the gastric wall, particularly those caused by gas-producing bacteria, resulting in the formation of gas bubbles. It represents a subset of phlegmonous gastritis and is frequently observed in patients with severe underlying conditions and sepsis, contributing to a high mortality rate^15,17,18^. Although we initially planned to analyze patients with emphysematous gastritis as well as those with gastric emphysema, we abandoned this plan because there was only one patient with emphysematous gastritis. In this one patient, the histopathological analysis of the resected stomach revealed phlegmonous inflammation of the stomach walls. Since none of the patients in the present study underwent cultures of gastric tissue or fluid, some patients may have been misdiagnosed with gastric emphysema despite the actual correct diagnosis being emphysematous gastritis. However, the diagnostic criteria for emphysematous gastritis have not been established^18^. In some previously reported cases, emphysematous gastritis was diagnosed without culture testing^19^. Further investigation is required to determine methods for distinguishing between these two conditions and whether they are truly distinct disease entities.

This study had several limitations. First, some patients with emphysematous gastritis may have been categorized as having gastric emphysema because of the lack of cultures. The possibility of emphysematous gastritis cannot be disregarded in patients with sepsis or those undergoing antibiotic treatment. As mentioned earlier, the diagnostic criteria for emphysematous gastritis have not been established; therefore, only one case diagnosed histologically as emphysematous gastritis was excluded from this study. Establishing a clear criteria to differentiate between emphysematous gastritis and gastric emphysema is necessary in future studies. Second, the selection of treatment modalities and scheduling of subsequent evaluations were determined at the discretion of attending physicians across diverse healthcare institutions. Particularly, as the timing of CT scans for confirming the resolution of intramural gas varied depending on the individual cases, gastric emphysema may have shown improvement at an earlier stage than the results obtained in this study (17.1 ± 34.9 days). Third, the implementation rate of endoscopic examinations was less than half number of the enrolled patients (18/45, 40.0%). However, given the rarity of gastric emphysema, the compilation of 18 cases in this study represents the most extensive dataset available to date. Notably, an analysis of the endoscopic findings has not been previously reported. We believe that the endoscopic features identified in this analysis will enhance our understanding of this condition.

In conclusion, we analyzed the characteristics, endoscopic features, and outcomes of 45 patients with gastric emphysema. This study represents the first English-language report to analyze endoscopic findings in gastric emphysema, and we wish to emphasize the following observations: endoscopically, gastric emphysema manifested as redness, erosion, coarse mucosa, and ulcers, with fewer mucosal injuries on the anterior wall (72.2%), a clear demarcation between areas of mucosal injury and intact mucosa (61.1%), and predominant longitudinal mucosal injuries on the folds of the stomach (50.0%). Understanding these unique endoscopic findings may have diagnostic implications in this condition.

## Data Availability

The datasets generated and analyzed in the current study are available from the corresponding author upon reasonable request.

## Abbreviations

CT: Computed tomography
SD: Standard deviation

## References

[1] López-Medina G, Castillo Díaz de León R, Heredia-Salazar AC, Hernández-Salcedo DR (2014). Gastric emphysema a spectrum of pneumatosis intestinalis: A case report and literature review. Case Rep. Gastrointest. Med..

[2] Soon MS, Yen HH, Soon A, Lin OS (2005). Endoscopic ultrasonographic appearance of gastric emphysema. World J. Gastroenterol..

[3] Shipman PJ, Drury P (2001). Emphysematous gastritis: Case report and literature review. Australas. Radiol..

[4] Al-Jundi W, Shebl A (2008). Emphysematous gastritis: Case report and literature review. Int. J. Surg..

[5] Loi TH, See JY, Diddapur RK, Issac JR (2007). Emphysematous gastritis: A case report and a review of literature. Ann. Acad. Med. Singap..

[6] Iwamuro M (2018). Conservative management of gastric emphysema and hepatic portal venous gas: A case report. Nihon Shokakibyo Gakkai Zasshi.

[7] Iwamuro M (2020). Cytomegalovirus colitis followed by colonic pseudolipomatosis and gastric emphysema in a post-resuscitation patient. Intern. Med..

[8] Ihoriya H (2019). Gastric emphysema in a critically ill patient successfully treated without surgery. Case Rep. Crit. Care.

[9] Iwamuro M (2021). Clinical characteristics of five patients with gastric emphysema. Nihon Shokakibyo Gakkai Zasshi.

[10] Alataby H, Daniel M, Bibawy J, Diaz K, Nfonoyim J (2020). Gastric emphysema and hepatic portal vein gas as complications of noninvasive positive pressure ventilation. Cureus.

[11] Furihata T (2020). Non-surgical treatment of gastric emphysema with intraabdominal free gas and hepatic portal venous gas: Lessons from a rare case. SAGE Open Med. Case Rep..

[12] Muratsu A, Muroya T, Kishimoto M, Kuwagata Y (2019). Gastric emphysema with portal emphysema due to superior mesenteric artery syndrome developing septic shock: A case report. Acute Med. Surg..

[13] Parikh MP, Sherid M, Ganipisetti V, Gopalakrishnan V, Habib M, Tripathi M (2015). Vomiting-induced gastric emphysema and hepatoportal venous gas: A case report and review of the literature. Case Rep. Med..

[14] Akella J, Fuentes GD, Kaur S, Venkatram S (2011). Emphysematous pyelonephritis associated with emphysematous gastritis and air in the portal vein. Gastroenterol. Res..

[15] Tagliaferri A, Melki G, Mohamed A, Cavanagh Y, Grossman M, Baddoura W (2023). Gastric pneumatosis in immunocompromised patients: A report of 2 cases and comprehensive literature review. Radiol. Case Rep..

[16] Inayat F, Zafar F, Zaman MA, Hussain Q (2018). Gastric emphysema secondary to severe vomiting: A comparative review of 14 cases. BMJ Case Rep..

[17] Watson A, Bul V, Staudacher J, Carroll R, Yazici C (2017). The predictors of mortality and secular changes in management strategies in emphysematous gastritis. Clin. Res. Hepatol. Gastroenterol..

[18] Bak MA, Rajagopalan A, Ooi G, Sritharan M (2023). Conservative management of emphysematous gastritis with gastric mucosal ischaemia: A case report. Cureus.

[19] Ng CY, Hayati F, Nadarajan C (2020). Emphysematous gastritis after metastatic malignant melanoma: A radiological surprise. BMJ Case Rep..

